# ApoE Inhibits the Progression of Glioma by Activating Immune Function

**DOI:** 10.1111/jcmm.70697

**Published:** 2025-07-15

**Authors:** Xiao‐fei Liu, Yu‐jie Chang, Min Long, Kai‐qi Huang, Bing Wang, Duo Gong, Jun‐Li Luo, Yong Feng

**Affiliations:** ^1^ Hengyang Medical School University of South China Hengyang Hunan China; ^2^ Department of Neurosurgery The Second Affiliated Hospital, Hengyang Medical School, University of South China Hengyang Hunan China; ^3^ Institute of Cardiovascular Disease, Key Laboratory for Arteriosclerology of Hunan Province Hengyang Medical School, University of South China Hengyang Hunan China; ^4^ The Cancer Research Institute and the Second Affiliated Hospital Hengyang Medical School, University of South China Hengyang Hunan China; ^5^ National Health Commission key Laboratory of Birth Defect Research and Prevention Hunan Provincial Maternal and Child Health Care Hospital Changsha Hunan China; ^6^ Institute for Future Sciences, University of South China University of South China Changsha Hunan China; ^7^ MOE Key Lab of Rare Pediatric Diseases & Institute of Otorhinolaryngology, Head and Neck Surgery University of South China Changsha Hunan China

**Keywords:** ApoE, CD8^+^ T cell, glioma, immunity, tumour suppressor

## Abstract

Glioma, marked by a low mutational burden, low immunogenicity, high heterogeneity, and the challenges posed by the blood–brain barrier, continues to be a major hurdle in neuro‐oncology. Current research underscores the necessity for more effective medications and treatment strategies. In this study, we explored the role of Apolipoprotein E (ApoE) in glioma using both bioinformatics and experimental methods. The construction of our bioinformatics risk model identified ApoE as a protective factor linked to longer survival in glioma patients. Subsequently, we created an in situ tumorigenic mouse model and a subcutaneous tumorigenic mouse model with ApoE gene knockout to evaluate the functional impacts of ApoE deficiency in glioma. Our results demonstrated that ApoE deficiency accelerates the growth of glioma and encourages the invasive behaviour of tumour cells into normal brain tissue. Additionally, we detected a reduction in the immune surveillance of glioma in the context of ApoE deficiency. Furthermore, flow cytometry analysis indicated that the lack of ApoE led to a decrease in positive immune cells and an increase in immunosuppressive cells within the tumour microenvironment. Our findings suggest that ApoE plays a crucial role in modulating glioma progression and immune surveillance, highlighting its potential as a therapeutic target.

## 1 Introduction

Glioma stands as the most common primary brain tumour among adults [1]. Glioma stands as the most common primary brain tumour among adults. Not only is it highly invasive, but it also features diffuse infiltration, indistinct boundaries, and aggressive proliferation [2]. Thanks to the advancements in molecular biological techniques, our comprehension of the pathogenesis of glioma has been significantly enhanced. Clinically, key genetic alterations have been identified. However, despite the remarkable improvements in the diagnosis and treatment of gliomas, the prognosis for glioma patients remains bleak, with the terribly poor five‐year survival rate. In the case of glioblastomas (GBMs), the median survival period is merely around 15 months [3, 4].

Tumour heterogeneity is a defining characteristic of glioma, which complicates the effectiveness of drugs targeting single targets or cellular populations. Even when patients have similar pathological and molecular profiles, treatment outcomes can vary significantly because of this heterogeneity. Glioma is characterised by low immunogenicity, with only 2% of gliomas having both TERT and IDH mutations, and 7% having only IDH mutations [5]. Compared with other solid tumours, the low mutation burden in gliomas makes them difficult for immune cells to recognise and eliminate effectively. Furthermore, most of the infiltrating immune cells in gliomas are immunosuppressive, with a relatively low proportion of CD8^+^ T cells and a marked increase in M2‐type macrophages [6, 7].

Treatment options for gliomas are limited, and their effectiveness is also somewhat restricted [8]. Surgery is the go‐to approach for maximising tumour removal. However, since gliomas frequently infiltrate the surrounding brain tissues, surgeons usually can only achieve partial tumour resection. Radiotherapy provides local treatment advantages by controlling tumour growth [9]. Chemotherapy and targeted therapy, which are widely used in cancer treatment, yield less‐than‐optimal results in gliomas. Chemotherapy drugs have difficulty penetrating the blood–brain barrier, thus impeding their access to tumour cells [10]. Meanwhile, the heterogeneity of gliomas also undermines the effectiveness of some targeted therapies [11]. Therefore, enhancing treatment efficacy and survival rates remains a major challenge in the clinical management of glioma patients. Current research focuses on developing more effective drugs and treatment strategies, including progress in immunotherapy, gene therapy, and improving the delivery efficiency of existing treatments. At present, research on glioma mainly focuses on the proliferation and invasion of glioma cells. Currently, the hot topic in glioma research is the changes in the glioma microenvironment.

Apolipoprotein E (ApoE), a secreted glycoprotein, is a remarkable molecule that exerts diverse pleiotropic effects on metabolism and immunity within the organism [12]. Growing evidence indicates that both tumour‐derived and stroma‐derived ApoE play a significant role in the battle against melanoma. It seems ApoE can suppress the progression of melanoma and influence the metastatic cascade. Specifically, it inhibits the invasiveness of melanoma cells and restricts their ability to recruit endothelial cells, which is vital for metastasis. Studies have shown that overexpressing certain microRNAs that silence ApoE in metastatic melanoma cells promotes metastasis formation [13]. This clearly underscores how suppressing ApoE may fuel cancer progression.

In stark contrast to tumour‐derived ApoE, which often gets repressed as melanoma progresses, the expression of stroma‐derived ApoE expression is significantly influenced by host genetic factors [14]. The presence of stroma‐derived ApoE plays a crucial part in anti‐tumour immunity by carefully modulating the populations of myeloid immune cell [15]. This reveals a dual role for ApoE: on one hand, it might promote tumour growth, while on the other, it has the potential to enhance immune responses against tumours. This situation highlights the critical balance between tumour‐ and host‐derived ApoE in determining its overall impact on cancer development, especially regarding proliferation and metastasis. To fully grasp the mechanisms and explore potential therapeutic opportunities related to ApoE modulation in cancer treatment, further in‐depth research is urgently needed.

Utilising techniques such as flow cytometry, we delved into the role of ApoE in the glioma microenvironment. Our results indicate that the absence of ApoE remarkably speeds up glioma growth and encourages the invasive nature of tumour cells. Additionally, knocking out ApoE has a notable impact on the infiltration and cytotoxic capabilities of CD8^+^ T cells.

## 2 Materials and Methods

### 2.1 Data Acquisition and Prognostic Signature Construction

Glioma tumour samples were sourced from the TCGA database (https://portal.gdc.cancer.gov/) and the Chinese Glioma Genome Atlas (CGGA, http://www.cgga.org.cn/). Genes identified with a *p*‐value < 0.05 through univariate COX regression analysis were selected for LASSO‐COX regression analysis. Stepwise multivariate COX regression analysis was conducted using the “survival” R package. A risk score formula was created to calculate the likelihood of poor survival for each sample, utilising the expression levels of prognostic genes weighted by their respective coefficients from the multivariate analysis.

### 2.2 Evaluation of Overall Survival (OS) Rates

Glioma patients from the CGGA and TCGA databases were classified into high‐risk and low‐risk subgroups based on the median risk score. Risk factor maps were visualised using “ggplot2” and “pheatmap” R packages. Survival analysis, using the “survival” and “survminer” R packages, assessed survival differences between the groups to validate the OS risk prognostic signature. Kaplan–Meier method estimated OS probability, with significance tested by the log‐rank test (*p* < 0.05). The “timeROC” R package generated ROC curves to assess sensitivity and specificity, and decision curve analysis (DCA) further validated the model. Univariate and multivariate Cox analyses confirmed the risk score's independent prognostic value, visualised by the “forestplot” R package. Correlations between risk scores and clinical characteristics (grade and IDH status) were shown using the “ggpubr” R package.

### 2.3 Bioinformatics Analysis

The Adult‐GCCA database was selected via the online platform GlioVis (https://gliovis.bioinfo.cnio.es/), and the correlations between ApoE and TSC2, ATRX, BRAF, MET, PRF1, GZMA, and GZMB were analysed. Single‐cell transcriptome data of gliomas were analysed using TISCH (http://tisch.compbio.cn/home/) to identify the cell types in which ApoE is predominantly expressed. Analysis demonstrated the correlation between ApoE expression in glioblastoma multiforme (GBM) and macrophage infiltration levels via the TIMER2.0 website (http://timer.cistrome.org/).

### 2.4 Cell Culture and Treatment

GL261 murine malignant glioma cell lines were sourced from Abiowell (AW‐CCM392) and cultured in Dulbecco's Modified Eagle Medium (DMEM, Procell, PM150210). The medium was supplemented with 1% Penicillin–Streptomycin Solution (Procell, PB180120), and 10% Fetal Bovine Serum (FBS, Procell, 164210‐50).

The 293 T cell lines, human glioma U251 cell lines, and human glioma Ln229 cell lines were cultured in Dulbecco's Modified Eagle Medium (DMEM, Procell, PM150210). The ApoE‐OE plasmid and OE‐NC plasmid were constructed by Beijing Tsingke Biotech Co. Ltd. and their authenticity was verified through bacterial liquid sequencing. Subsequently, the OE‐NC plasmid and ApoE‐OE plasmid were co‐transfected into 293T cells along with the packaging plasmids psPAX2 and pMD2.G at a mass ratio of 4:3:2 to generate a lentivirus. The lentivirus was then used to infect into U251 and Ln229 cells for a duration of 48 h. Following the infection, the cells were subjected to selection using puromycin to obtain cell populations that stably and highly expressed ApoE.

### 2.5 Real‐Time qPCR

Reverse transcription was conducted using the TAKARA reverse transcription kit (Takara, Dalian, China) as per the manufacturer's instructions. Real‐time PCR was carried out in the Bio‐Rad CFX Maestro (Hercules, USA) using PCR master mix (Sango Biotech, Shanghai, China). The primers used were as follows: for SLC25A10, forward primer 5′‐ACCTGCTCAAGGTGCATCTG‐3′ and reverse primer 5′‐CAGGGAGTAGGTCATCTGTCTG‐3′; for HEXA, forward primer 5′‐ACGTCCTTTACCCGAACAACT‐3′ and reverse primer 5′‐CGAAAAGCAGGTCACGATAGC‐3′; for MBOAT1, forward primer 5′‐GTTTCGCATCTACTTACGTCCTG‐3′ and reverse primer 5′‐GCACATTAACACCAGCACAAAA‐3′; for ApoE, forward primer 5′‐GTTGCTGGTCACATTCCTGG‐3′ and reverse primer 5′‐GCAGGTAATCCCAAAAGCGAC‐3′; for ACOT7, forward primer 5′‐TCTCCCATGTGCATCGGTG‐3′ and reverse primer 5′‐TTTTCGGACATCACGTTGACC‐3′; for CPNE6, forward primer 5′‐CCCCGAAATGATACCTTCCTCG‐3′ and reverse primer 5′‐CTTAGTGACCTTGGTTTGTGACA‐3′; for Arg1, forward primer 5′‐CTCCAAGCCAAAGTCCTTAGAG‐3′ and reverse primer 5′‐AGGAGCTGTCATTAGGGACATC‐3′; for Adora2, forward primer 5′‐GCCATCCCATTCGCCATCA‐3′ and reverse primer 5′‐GCAATAGCCAAGAGGCTGAAGA‐3′; for Tac1, primer 5′‐CAGTCACCAACTCAGTCCTGC‐3′ and reverse primer 5′‐CACAACGATCTCGAAGTCCCC‐3′; for Lrp1, primer 5′‐GCTGGGGTGTACGGAAATGG‐3′ and reverse primer 5′‐GTGCTCGAATTTGTTCTGGACT‐3′. GAPDH served as an internal control, with forward primer 5′‐CTGGGCTACACTGAGCACC‐3′ and reverse primer 5′‐AAGTGGTCGTTGAGGGCAATG‐3′.

### 2.6 Western Blotting

The protein concentration in whole cell lysate was quantified using the Bicinchoninic acid assay. Equal amounts of total proteins were mixed with 5 × loading buffer containing 10% β‐mercaptoethanol. The mixtures were heated at 100°C for 10 min, and then, applied to a 10% SDS‐polyacrylamide gel (10 μg of total proteins per well). After electrophoresis, the proteins were transferred onto polyvinylidene difluoride (PVDF) membranes. The PVDF membranes were blocked with 5% non‐fat dry milk (NFDM) for 2 h at room temperature. Following the blocking step, the membranes were incubated overnight at 4°C with primary antibodies specific to the corresponding proteins or GAPDH. After the overnight incubation, the membranes were washed three times with TBST and incubated with secondary antibodies for 1 h at room temperature. After another three‐time wash with TBST, antibody binding was visualised with ECL Plus. The membranes were scanned using Image Lab software, and semi‐quantitative analysis was performed by comparing the signals of the target proteins with that of GAPDH.

### 2.7 Cell Proliferation

U251‐OE‐NC, U251‐ApoE‐OE, Ln229‐OE‐NC, and Ln229‐ApoE‐OE cells in logarithmic growth phase were digested using 0.25% trypsin and then counted. A 96‐well plate was utilised, with five wells allocated for each group. Each well was inoculated with 1000 cells in a volume of 150 μL. The outer‐edge wells of the plate were filled with 150 μL of PBS buffer. The culture plate was then incubated overnight in a constant‐temperature incubator maintained at 37°C, with a 5% CO_2_ atmosphere and saturated humidity. On the first, third, and fifth day after the overnight incubation, 15 μL of MTT was added to each well. The plate was then returned to the constant‐temperature incubator under the same conditions (37°C, 5% CO_2_, saturated humidity) for 3–4 h. After this incubation period, the culture medium was removed, and 150ul of DMSO was added to each well. The plate was further incubated in the same incubator for 15 min. Following a 5‐s shaking of the plate in an ELISA reader, the OD value at 570 nm was measured for each well. The well containing only the culture medium without any inoculated cells served as the blank zeroing well.

### 2.8 Cell Scratch Experiment

U251‐OE‐NC, U251‐ApoE‐OE, Ln229‐OE‐NC, and Ln229‐ApoE‐OE cells in the logarithmic growth phase were digested with 0.25% trypsin and counted. A 12‐hole plate was selected, and 3–4 evenly‐sized horizontal lines were drawn in parallel and at equal intervals using a black marker pen on the back of the plate. 5 × 10^4^ target cells in the logarithmic growth phase were inoculated into the 12‐well plate, ensuring uniform distribution of the cells. Incubate overnight in a constant temperature incubator at 37°C, 5% CO_2_, and saturated humidity. After removing the 12‐well plate from the incubator, a sterile 200 μL pipette tip was used to draw two parallel and evenly‐spaced vertical lines perpendicular to the pre‐drawn horizontal lines. The wells were washed 2–3 times with PBS buffer to remove as many floating cells as possible. The medium was then replaced with serum‐free medium, and the cells were cultured for 15–30 mins. Pictures were taken under a microscope, and this time point was recorded as 0 h. Based on the inherent growth rate of the cells, pictures were taken again 24 h after the scratching and recorded. The migration distance of cells at different time points was calculated at the same positioning point. For each well, the width of the cell migration distance was measured at least at 4–6 locations, and the average value was taken.

### 2.9 Subcutaneous and In Situ Glioma Models

For subcutaneous injection, GL261 cells were harvested via trypsinization (Gibco) and adjusted to a concentration of 1 × 10^5^ cells per μL. Male wild‐type mice and ApoE knockout mice were subcutaneously (interscapularly) injected with 1 × 10^6^ cells in a total volume of 100 μL. The mice were sacrificed 30 days after tumour transplantation.

For intracranial injection, GL261 cells were collected through trypsinization (Gibco) and brought to a concentration of 1 × 10^5^ cells per μL for injection. Briefly, male wild‐type mice and ApoE knockout mice were anaesthetised using 10% ethyl carbamate at a dose of 0.10 uL per 10 g of body weight and then immobilised in a stereotactic frame. A midline incision was made on the skin to expose the scalp. A microdrill was used to perform a craniotomy 2‐mm anterior and 1.5‐mm lateral to the bregma. Subsequently, 5 × 10^5^ cells in a total volume of 5 μL were slowly injected over 1 min at a depth of 3 mm with a microsyringe.

### 2.10 Co‐Culture

A 6‐well trans‐well chamber with 0.4 μm pore polycarbonate membrane (Corning, USA) was utilised in this study. For co‐culture of peritoneal macrophages and lymphocytes, ApoE knockout mice and wild‐type mice were intraperitoneally injected with 1 mL of 3% mercaptoacetate broth for three consecutive days. Peritoneal macrophages were harvested by peritoneal washing with PBS (1500 rpm, 5 min). Then peritoneal macrophages were added in the upper chamber, and spleen lymphocytes were added in the lower chamber. The cells were collected and analysed after 48 h co‐culture. Experiments were performed in triplicate.

### 2.11 Flow Cytometry

Antibodies for PE‐Cyanine7 anti‐mouse CD3 (Biolegend, Cat#100220), APC‐Cyanine7 anti‐mouse CD45 (Biolegend, Cat#103105), PerPCP‐Cy5.5 anti‐mouse CD4 (Biolegend, Cat#100434), FITC anti‐mouse CD8 (Biolegend, Cat#100705), BV421 anti‐mouse PD‐1 (Biolegend, Cat#135218), BV711 anti‐mouse LAG3 (Biolegend, Cat#125219), PE anti‐mouse Perforin (Biolegend, Cat#154306), and Alexa Fluor 647 anti‐mouse Granzyme B (Biolegend, Cat#515406) were obtained from Biolegend. Flow cytometry analyses were performed using the Cytek Athena instrument, and the results were evaluated with FlowJo software.

### 2.12 Human Glioma Samples

Formalin‐fixed, paraffin‐embedded glioma tissue samples, along with encoded clinicopathological data and follow‐up information, were collected from 169 patients treated at The Second Affiliated Hospital of University of South China between June 2010 and December 2016. The Research Ethics Committee of Second Affiliated Hospital of University of South China approved the studies.

### 2.13 Immunohistochemical Staining

Immunohistochemical staining was performed as described previously. Briefly, serial 4 μm‐thick glioma tissue sections were prepared. After deparaffinisation and rehydration, slides were subjected to antigen retrieval using Tris‐EDTA (pH 9.0) buffer. They were then blocked with 3% H_2_O_2_ solution for 10 min, followed by 1‐h incubation with 5% goat serum, and then incubated overnight at 4°C with ApoE primary antibodies. Subsequently, the slides were treated with Two‐Step IHC reagents and 3,3‐diaminobenzidine (DAB) solution according to the manufacturer's instructions. Harris' modified haematoxylin was used for counterstaining. Staining intensity in cancer cells was categorised as negative (0), weak (1), moderate (2), and strong (3), while the percentage of positive cells was defined as 0 (0%–5%), 1 (5%–25%), 2 (26%–50%), 3 (51%–75%), or 4 (> 75%). The total score for each sample was obtained by multiplying the staining intensity and positive cell scores. A total score < 5 indicated high ApoE expression, while a score < 5 indicated low ApoE expression. The immunological statistical results are shown below.

### 2.14 Statistical Analysis

All data were presented as mean ± SEM and evaluated using Two‐tailed, unpaired Student's *t*‐tests and oneway ANOVA. A value of *p < 0.05, **p < 0.01, ***p < 0.001 was considered statistically significant. All the statistical analyses were performed using GraphPad Prism 8 software. All experiments were performed at least three independent times.

## 3 Results

### 3.1 Identification of the Prognostic Signature by Risk Scores in Glioma

**FIGURE 1:**
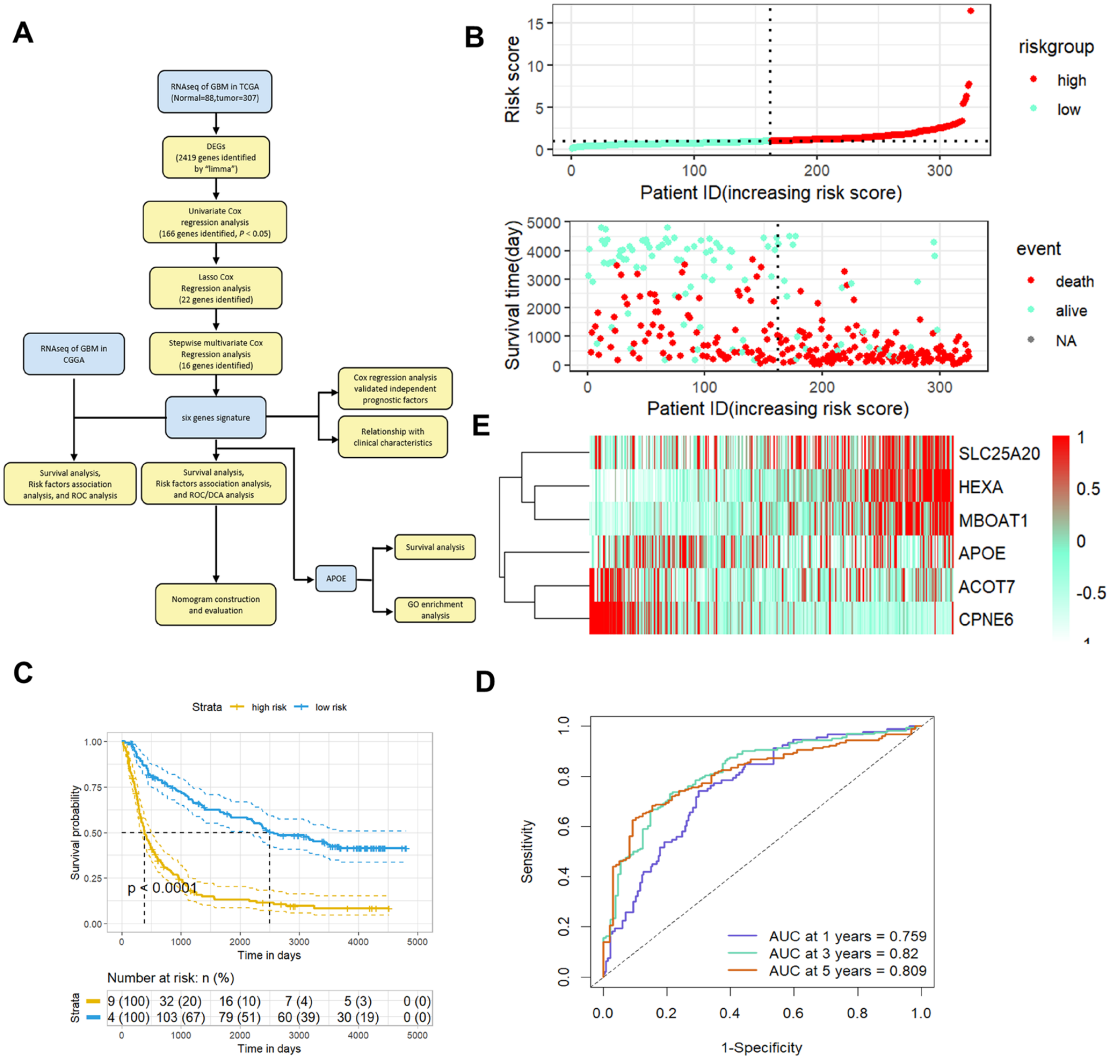
(A) Workflow diagram of this study. This flowchart depicts the systematic identification and validation of a 6‐gene signature for the prognostic prediction of glioma. Identification and validation of risk score based on gene signature of glioma patients in Chinese Glioma Genome Atlas (CGGA). (B) Risk plot where each point, corresponding to an individual patient, is arranged according to the risk score. Green represent patients with low‐risk scores, while red points denote those with high‐risk scores, respectively. (C) Kaplan–Meier analysis of glioma patients in CGGA, stratified by the median risk. High risk scores are associated with poorer survival outcomes. (D) Receiver Operating Characteristic (ROC) demonstrating the performance of the risk score in predicting the prognosis of glioma patients in the CGGA. (E) Distribution of the risk score and the significant genes associated with glioma in the CGGA.

To comprehensively explore the prognostic signature in glioma, we meticulously analysed the datasets of glioma samples, which consisted of 307 cases retrieved from the TCGA database. Concurrently, we also included 88 non‐tumour samples from the GTEx for comparison. After performing a series of rigorous analyses, including univariate Cox regression, LASSO‐COX regression, and multivariate Cox regression, we successfully established the gene signature (Figure 1A).

We stratified patients into low‐ and high‐risk scores groups and constructed association maps. These maps vividly revealed that as the curve of the risk score ascended, the mortality rate of glioma patients increased accordingly (Figure 1B). Subsequently, we carried out the Kaplan–Meier logarithm test to evaluate the overall survival (OS) disparities between the different risk score groups. The results clearly indicated that patients with high‐risk scores experienced poorer survival outcomes compared to those with low‐risk scores (Figure 1C). We then utilised the receiver operating characteristic (ROC) curves to gauge the predictive efficiency of the OS‐related signature. The area under the curve (AUC) values for 1‐year, 3‐year, and 5‐year were 0.759, 0.82, and 0.809 respectively. These values strongly suggest that the signature possesses excellent predictive capabilities (Figure 1D).

Subsequently, we carefully screened 16 relevant genes to create a prognostic signature. It is worth noting that this signature encompasses genes such as SLC25A20, HEXA, MBOAT1, APOE, ACOT7, and CPNE6 (Figure 1E).

### 3.2 The Relationship Between ApoE and the Prognosis of Glioma

**FIGURE 2:**
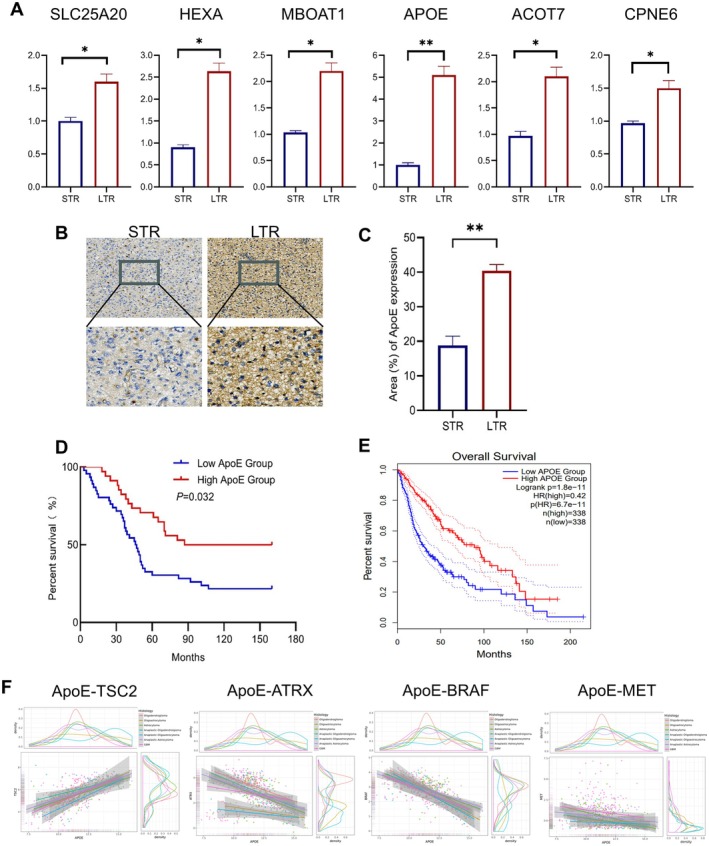
Correlation of ApoE with overall survival and molecular functions in glioma patients. (A) qPCR analysis was conducted to compare 6 genes between two groups of glioma patients: Those with a long time to recurrence (> 6 months, LTR) and those with a short time to recurrence (≤ 6 months, STR). (B, C) Dichromatic immunohistochemical (IHC) staining was performed on ApoE in the LTR and STR gruops. (B) Representative images of the dichromatic IHC staining are presented. (C) Quantification of the staining results was carried out. (D) Kaplan–Meier survival analysis of ApoE in glioma patients from our cohort was performed. (E) Using data from The Cancer Genome Atlas (TCGA) dataset, a Kaplan–Meier survival analysis of ApoE in glioma patients was conducted. Patients were divided into low‐ and high‐ApoE expression groups based on the median expression level. (F) On the Gliovis (https://gliovis.bioinfo.cnio.es/) website, within the CGGA database, the Pearson correlation between ApoE and common glioma related genes (ATRX, MET, TSC2, BRAF) was analysed. The results are displayed as mean ± SEM. Significance levels are noted as *p < 0.05, **p < 0.01.

We collected 80 glioma patients and analysed their clinical data. The results showed that ApoE expression was lower in high‐grade gliomas compared with fappedlow‐grade gliomas (Table 1). To further explore the correlation between gene expression and glioma progression, we collected specimens from 15 patients, dividing them into two groups: those with a long time to recurrence (LTR > 6 months) and those with a short time to recurrence (STR ≤ 6 months), for quantitative gene testing. We discovered that, out of these six genes (SLC25A20, HEXA, MBOAT1, APOE, ACOT7, and CPNE6), ApoE exhibited the most pronounced change in expression (Figure 2A). As a result, in the subsequent phase, we intend to carry out a thorough investigation into the role and underlying mechanism of ApoE within glioma.

**TABLE 1:** Baseline characteristics of glioma patients.

Characteristics	ApoE	Overall expression		*p*
		Low (*n* = 46)	High (*n* = 34)	
Age (*n*, %)				
≤ 48	40 (50)	21 (45.7)	19 (55.9)	
> 48	40 (50)	25 (54.3)	15 (44.1)	0.498
Sex (*n*, %)				
Male	48 (60)	28 (60.9)	20 (58.8)	
Female	32 (40)	18 (39.1)	14 (41.2)	1.000
WHO grade (*n*, %)				
Low (I&II)	35 (43.8)	9 (19.6)	26 (76.5)	
High (III&IV)	45 (56.2)	37 (80.4)	8 (23.5)	< 0.001
Ki67	18.1	23.6	10.5	0.003

We utilised immunohistochemistry to conduct a more comprehensive evaluation of ApoE expression in glioma patients. As depicted in Figure 2B,C, ApoE expression is significantly higher in LTR (> 6 months) compared to STR (≤ 6 months). Glioma patients who present with low ApoE expression tend to have a poorer overall survival rate (Figure 2D). This finding is consistent with the data obtained from glioma patients in the TCGA dataset (Figure 2E). By delving into the CGGA database to analyse the correlations between ApoE and genes associated with glioma growth and resistance, we found that ApoE has a positive correlation with TSC2, a well‐recognised tumour suppressor gene. In contrast, it shows a negative correlation with ATRX, a gene linked to TMZ resistance, and also with tumour‐growth‐promoting genes like BRAF and MET (Figure 2F). These findings strongly imply that ApoE may become a potential target for the treatment of glioma.

### 3.3 The Impact of ApoE Deficiency on Subcutaneous Glioma Tumorigenesis

**FIGURE 3:**
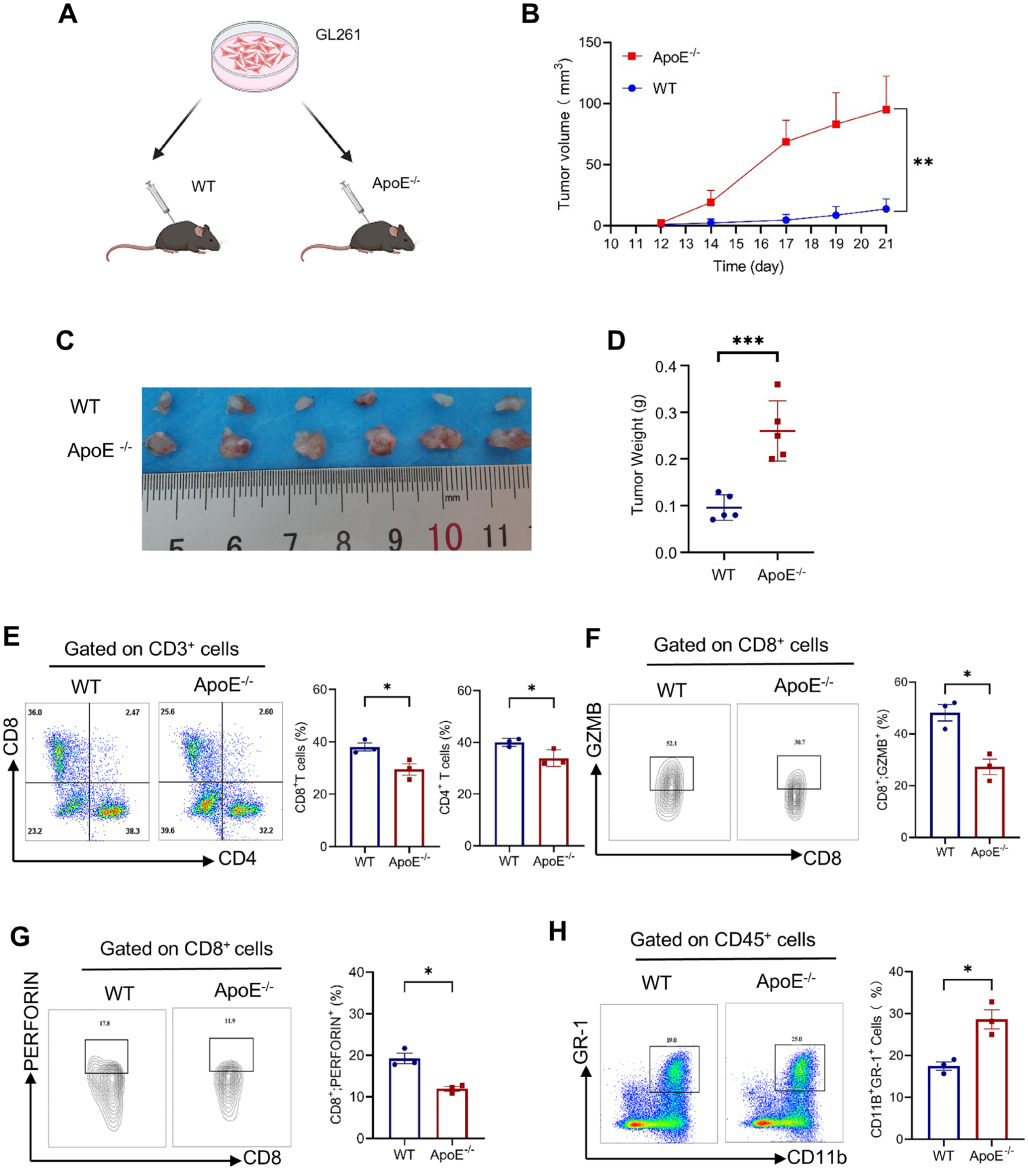
The influence of ApoE on mice with subcutaneous gliomas. (A) A schematic diagram is presented to outline the experimental setup comprehensively. (B–D) The mean growth of GL261 subcutaneous tumours in WT and ApoE^−/−^ mice (*n* = 5 per group) was investigated. The growth curve is shown in panel (B), representative tumour images are presented in panel (C), and the tumour weights are provided in panel (D). (E–G) Fluorescence‐activated cell sorting (FACS) was employed to analyse the proportions of CD8^+^ (E), GZMB^+^CD8^+^ (F) and Perforin^+^CD8^+^ (G) cells within the CD3^+^ tumour‐infiltrating lymphocytes (TILs) from ApoE^−/−^ or Wild‐Type (WT)mice, followed by quantification of the results. (H) FACS was used to assess the presence of CD11b^+^ and GR‐1 cells within CD45^+^ TILs from ApoE^−/−^ or WT mice. The results are presented as the mean ± SEM. Statistical significance levels are indicated as *p < 0.05, **p < 0.01, ***p < 0.001.

To investigate the effect of ApoE deficiency on tumour growth in subcutaneous tumours (Figure 3A), we conducted a series of experiments. In ApoE‐knockout (ApoE^−/−^) mice, glioma tumours demonstrated an accelerated growth rate (Figure 3B–D). There was a decrease in the infiltration of CD8^+^ T cells into the subcutaneous tumours of ApoE^−/−^ mice (Figure 3E). Flow cytometry analysis further revealed that in these ApoE‐knockout tumour‐bearing mice, both the circulating and intra‐tumoural levels of myeloid‐derived suppressor cells (MDSCs) were elevated (Figure 3F). This indicates that ApoE deficiency may create a more immunosuppressive microenvironment that promotes tumour growth.

### 3.4 The Effect of ApoE Deficiency on In Situ Glioma Tumorigenesis

**FIGURE 4:**
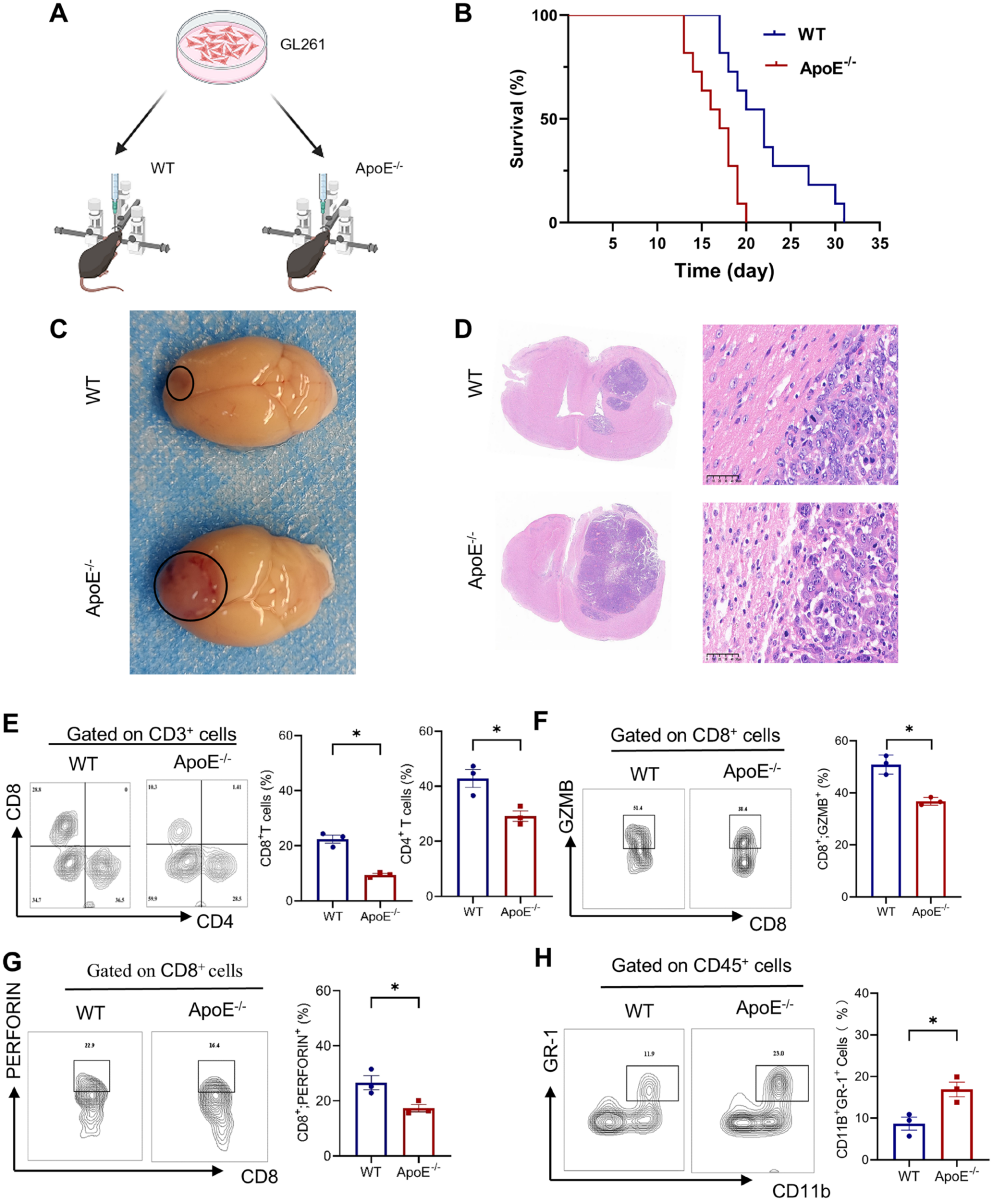
The impact of ApoE on mice with in situ glioma. (A) A schematic overview is provided to illustrate the experimental setup. (B) Kaplan–Meier survival analysis of WT and ApoE^−/−^ mice that were intracerebrally injected with GL261 cells (*n* = 7 per group); The log‐rank test was employed, and a highly significant difference was observed (*p < 0.0001). (C) The macroscopic appearance of the tumours was documented. (D) Haematoxylin and eosin (H&E) staining was performed on the tumour sections. (E–G) FACS was used to analyse the presence of CD8^+^ (E), GZMB^+^CD8^+^ (F) and Perforin^+^CD8^+^ (G) in CD3^+^ infiltrating lymphocytes (TILs) from ApoE^−/−^ or WT mice, followed by quantification. (H) FACS was also utilised to analyse CD11b^+^ and GR‐1^+^ cells within the CD45^+^ TILs from ApoE^−/−^ or WT mice, and the results were quantified. The results are presented as the mean ± SEM. Statistical significance levels are denoted as *p < 0.05.

Subsequently, we delved into the influence of the immune system on the growth behaviour of intracranial glioma (Figure 4A). To this end, we compared the survival rates and histomorphological features of GL261 glioma cell lines in C57BL/6 wild‐type (WT) mice and ApoE‐knockout (ApoE^−/−^) mice. When GL261 glioma cells were injected intracerebrally, ApoE^−/−^ mice exhibited a significantly shorter survival time compared to WT mice (median survival: 18 days for ApoE^−/−^ mice vs. 22 days for WT mice, *p* = 0.012) (Figure 4B). Macroscopic examination of the tumours revealed that the gliomas in ApoE^−/−^ mice was significantly larger in size than those in WT mice (Figure 4C). These results were consistent with the findings from our prior subcutaneous tumour formation studies. Furthermore, histological analysis corroborated the enhanced invasiveness of gliomas in ApoE^−/−^ mice (Figure 4D). This investigation provides valuable insights into the role of ApoE in modulating the immune‐tumour interaction within the intracranial microenvironment.

Similar to the results of previous subcutaneous tumour formation, in ApoE^−/−^ mice, we observed a decrease in CD8^+^ T cell infiltration (Figure 4E). Concurrently, the secretion of granzyme (Figure 4F) and perforin (Figure 4G) was diminished. In addition, we carried out detections of myeloid cell markers. Our findings indicated an increase in the number of myeloid‐derived suppressor cells (MDSCs) in ApoE^−/−^ mice (Figure 4H). These results further emphasise the role of ApoE in modulating the immune microenvironment of tumours, potentially influencing tumour progression and immune‐mediated anti‐tumour responses.

### 3.5 The Upregulation of ApoE Expression Exerts no Notable Influence on the Biological Behaviour of Glioma Cells

**FIGURE 5:**
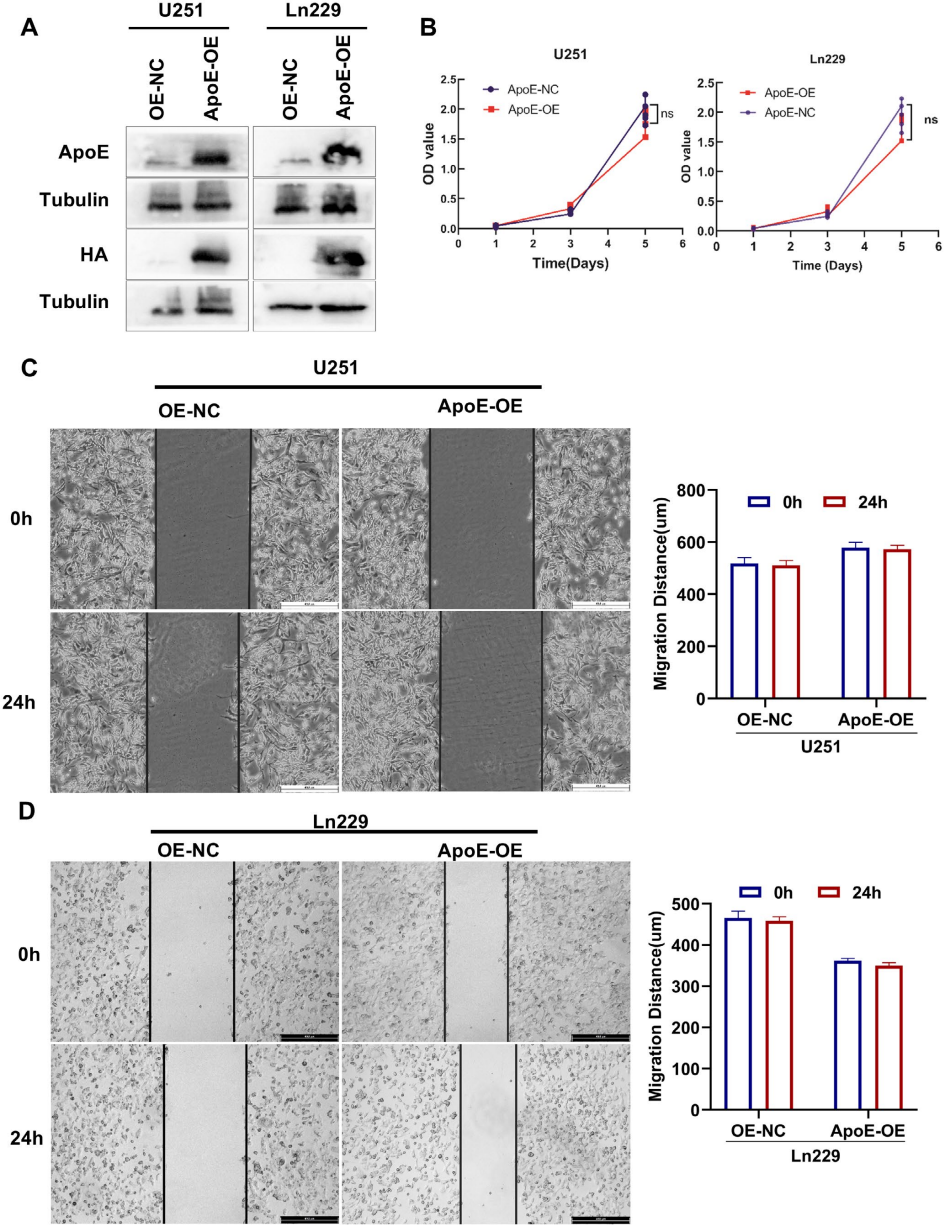
ApoE exerts no discernible impact on the proliferation and migration of glioma cell lines U251 and Ln229. (A) The western bloting assay was employed to examined the over‐expression of ApoE in the U251 and Ln229 cells. (B) The MTT assay was employed to delineate the proliferation curves of U251 and Ln229 cells. The results indicated no statistically significant difference (*p* > 0.05), suggesting that ApoE does not influence the proliferative capacity of these cells. (C, D) The wound‐healing assay was conducted to assess the influence of ApoE on the migratory potential of U251 cells (C) and Ln229 cells (D). The data revealed that ApoE had no significant effect on cell migration (*p* > 0.05).

To investigate whether ApoE has an impact on the biological behaviour of glioma cells, we overexpressed ApoE in U251 and Ln229 cell lines (Figure 5A). The MTT assay results revealed that the overexpression of ApoE did not exert a significant influence on the proliferation of U251 and Ln229 cell lines (Figure 5B). The outcomes of the wound‐healing assay indicated that the overexpression of ApoE had no marked effect on the migration of U251 and Ln229 cell lines (Figure 5C,D). This finding suggests that ApoE does not affect the proliferation and migration of glioma cells.

### 3.6 Analysis of Genes and Potential Signalling Pathways Associated With ApoE Knockout‐Induced Tumour Growth Promotion

**FIGURE 6:**
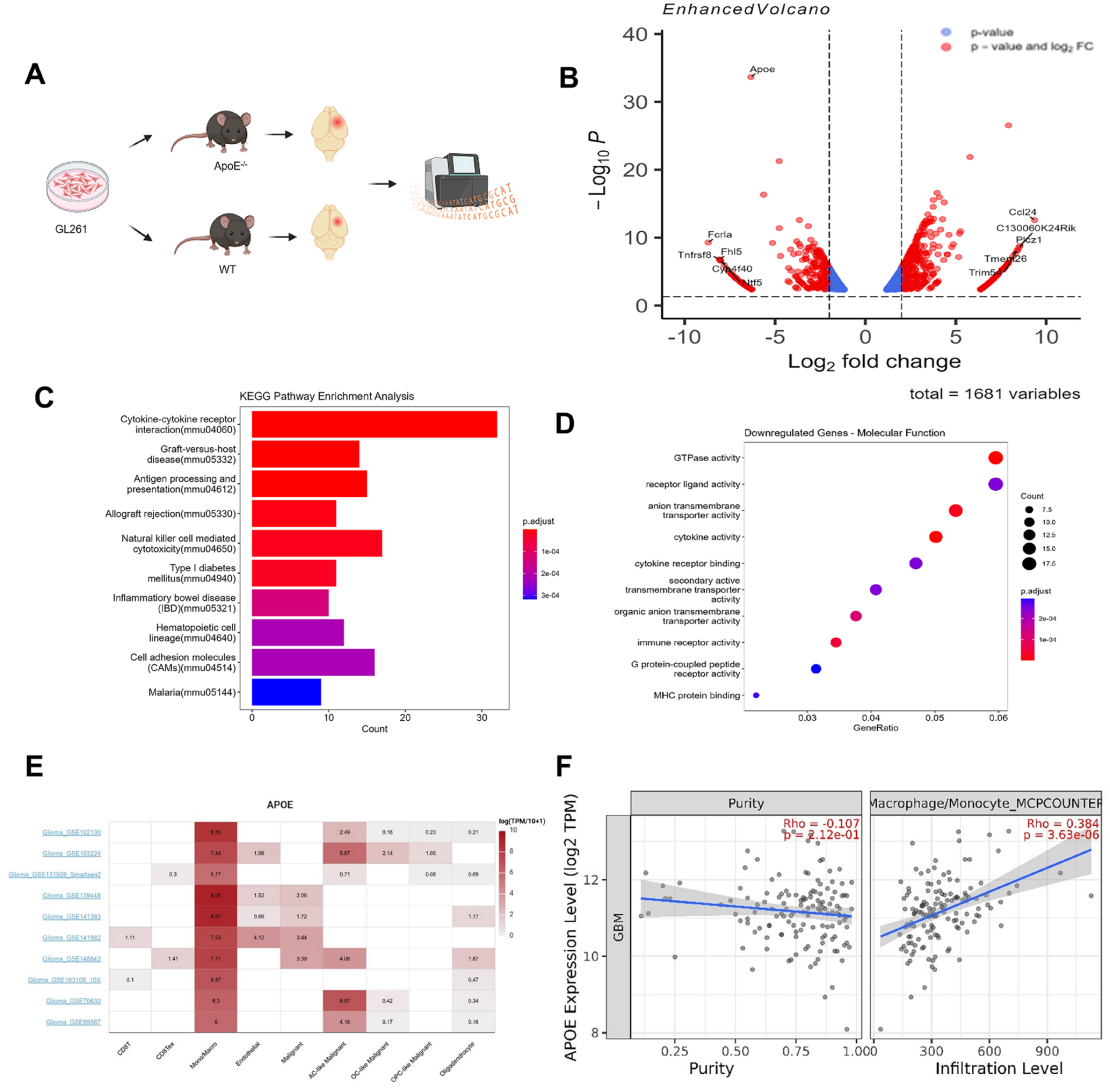
Analysis of the in situ tumorigenic transcriptome sequencing results in ApoE^−/−^ mice and WT mice. (A) Workflow for the Analysis of In Situ Tumour Tissues. (B) Volcano plot depicting differentially expressed genes across different groups. (C) Kyoto Encyclopedia of Genes and Genomes (KEGG) enrichment were carried out on 637 intersection genes. (D) Gene Ontology (GO) enrichment analyses were conducted on the same 637 intersection genes. (E) The TISCH website (http://tisch.comp‐genomics.org/documentation) was employed to analyse the primary cells expressing ApoE in glioma using single‐cell transcriptome sequencing data. (F) The TIMER2.0 website (http://timer.cistrome.org) analysis revealed a positive correlation between ApoE expression in glioma and tumour‐infiltrating monocytes/macrophages (*p* < 0.001).

To elucidate the underlying mechanism of ApoE in glioma, we carried out transcriptome sequencing on the tumour tissue and the paired contralateral non‐tumour brain tissue obtained from mice with orthotopic tumorigenesis. (Figure 6A). The transcriptome analysis results, as visualised by the volcano plots, indicated notable disparities in the gene expression profiles between the tumours of ApoE^−/−^ mice and those of WT mice. In the glioma tumour tissues of ApoE^−/−^ mice, the expression of ApoE was markedly decreased. Conversely, genes associated with antigen presentation, such as CCL24, and those related to T‐cell activation, like Lrr10b, were significantly upregulated (Figure 6B).

A comprehensive analysis of the four sets of differentially expressed genes indicated that the low expression of ApoE in tumours is linked to the regulation of the innate immune response (Figure 6C,D). Given that ApoE serves as a marker for macrophages and brain glial cells, the deficiency of ApoE may exert a substantial influence on antigen‐presentation function. This could potentially be associated with the massive infiltration of macrophages in the glioma. The GO enrichment analysis of differentially expressed genes produced comparable results. To elucidate the relationship between ApoE and macrophages, we examined the single‐cell transcriptome sequencing data of glioma retrieved from the TISCH database. Our analysis revealed that ApoE is predominantly expressed in macrophages/monocytes (Figure 6E). Likewise, within the TIMER2.0 database, the bulk transcriptome data demonstrated a strong positive correlation between ApoE expression and macrophages/monocytes (Figure 6F). These combined findings underscore the potential significance of ApoE in modulating the macrophage‐mediated immune response within the glioma microenvironment.

### 3.7 The Impact of Macrophage‐Specific ApoE Knockout on CD8 ^+^ T Cell Function

**FIGURE 7:**
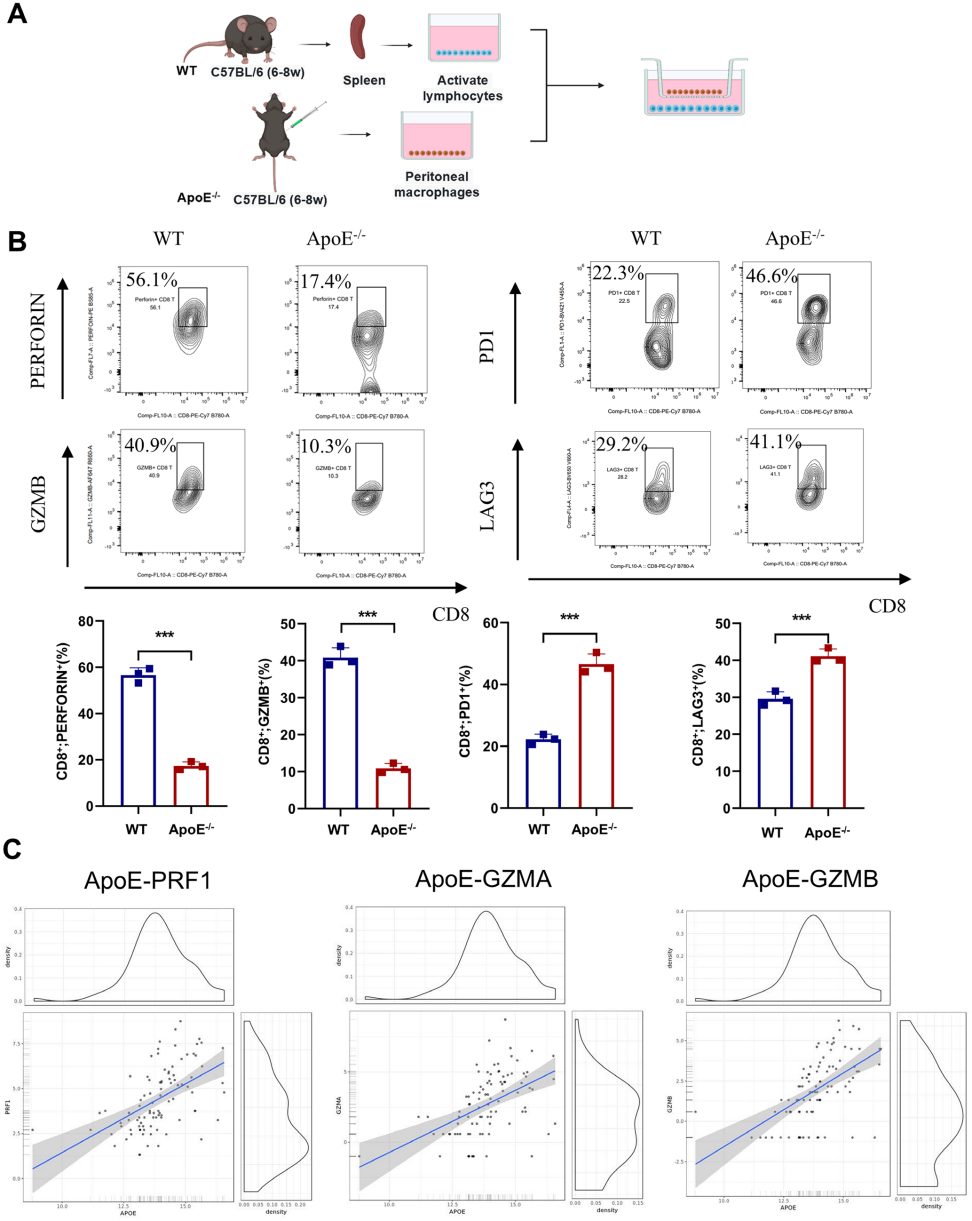
Macrophage‐specific knockout of ApoE significantly inhibits the tumour‐killing activity of CD8 T cells. (A) Schematic workflow of the co‐culture of peritoneal macrophages, T cells and GL261 cells. (B) Typical flow images of GZMB, PERFORIN, PD‐1 and LAG3 expression for each group of CD8+ T cells co‐cultured with GL261 cells and macrophages. (C) Utilising the Gill database on the Gliovis website (https://gliovis.bioinfo.cnio.es/), we analysed the Pearson correlation between ApoE and PERFOIN (PRF1), GZMA, and GZMB was analysed as our findings (*p* < 0.001).

Immunotherapies are emerging as a promising avenue that may offer new hope for the treatment of gliomas. To elucidate the influence of ApoE on regulating the macrophage‐mediated immune response in the glioma microenvironment, we set up a non‐contact co‐culture system. In this system, peritoneal macrophages obtained from ApoE^−/−^ and WT mice were co‐cultured with lymphocytes isolated from the spleens of WT mice (Figure 7A). Subsequently, flow cytometry was employed to analyse cell activation. The results indicated that, when compared to the WT mice group, macrophages lacking ApoE exhibited a decreased activation of positively‐functioning immune lymphocytes and an increased activation of negatively‐functioning immune lymphocytes (Figure 7B). This negative modulation of immune function could potentially be part of the ApoE‐mediated mechanism that acts against glioma. Similarly, t by conducting an analysis on the Gliovis website, we identified a strong positive correlation between ApoE and PERFORIN (PRF1), GZMA, and GZMB (Figure 7C). This observation implies that ApoE might play a role in promoting T‐cell activation and the expression of these proteins, consequently exerting an anti‐glioma effect.

## 4 Discussion

Gliomas stand as the most widespread primary malignant brain tumours globally, constituting a staggering 81% of central nervous system (CNS) malignancies. Alarmingly, these tumours often recur within six months post‐surgery [16, 17]. Nonetheless, the pursuit of effective glioma treatment encounters a significant roadblock in the form of the blood–brain barrier (BBB). This physiological barrier restricts the entry of effective medications into the brain, thereby posing formidable challenges to the development of anti‐glioma drugs [18, 19]. Consequently, there is an urgent need to explore novel and effective therapeutic approaches for glioma. In recent years, immunotherapeutic strategies brought about a paradigm shift in the treatment of various cancers. These innovative approaches also offer a glimmer of new hope for the management of glioma [20, 21].

In this study, we analysed the datasets of glioma samples (307 cases) from the TCGA database and non‐tumour samples (88 cases) from GTEx. We identified 16 relevant genes to develop a prognostic signature. Subsequently, we collected specimens from 15 patients with long‐time recurrence (time to recurrence > 6 months, LTR) and short‐time recurrence (time to recurrence ≤ 6 months, STR) for quantitative gene testing. The results revealed that 6 genes (SLC25A20, HEXA, MBOAT1, APOE, ACOT7, and CPNE6) were significantly up‐regulated in the LTR group. Among these genes, ApoE exhibited the most significant up‐regulation.

ApoE is a 35‐kDa protein composed of 299 amino acids, which is preceded by an 18‐amino‐acid N‐terminal endoplasmic reticulum (ER) signal peptide [22]. The protein encoded by the ApoE gene is ubiquitously expressed throughout the human body and possesses diverse biological functions, encompassing lipid metabolism, immune regulation, and cell signalling [22, 23]. Recent investigations have indicated that the ApoE gene might play a crucial role in the development and survival of glioma [24, 25]. Research on glioma has demonstrated that patients with high ApoE expression typically display longer survival times [13, 26, 27]. Immunohistochemical staining and quantification results indicated that ApoE expression was significantly upregulated in long‐term recurrence (LTR) group. Analysis revealed that glioma patients with low ApoE expression had poorer overall survival, which was consistent with the findings from glioma patients in the TCGA dataset. This observation implies that ApoE may impact the development of glioma and patient survival outcomes via multiple pathways. One proposed hypothesis is that high ApoE expression could be associated with decreased tumour cell invasion and an enhanced immune response. Through a combination of in vitro and in vivo experiments, we discovered that ApoE predominantly modulates the function of immune cells within the tumour microenvironment. It enhances the anti‐tumour immune response rather than directly regulating the proliferation, migration, and invasiveness of glioma cells, thus prolonging patient survival.

The tumour suppressor gene tuberous sclerosis complex‐2 (TSC2) has been demonstrated to directly inhibit cell growth and is highly expressed in normal central and astrocytes of the nervous system neurons [28]. In comparison with the control group, the frequency of TSC2 gene polymorphism was significantly elevated in gangliogliomas [29]. ATRX is a gene associated with temozolomide (TMZ) resistance. The BRAF inhibitor dabrafenib has exhibited significant clinical efficacy in paediatric patients with BRAF V600 mutation‐positive low‐grade glioma and high‐grade glioma [30]. Research has indicated that the mesenchymal‐epithelial transition factor (MET) plays a critical role in the proliferation, survival, migration, invasion, and therapeutic resistance and recurrence of glioblastomas [31] Bioinformatics predictions have shown that ApoE has a positive correlation with TSC2 and a negative correlation with ATRX, BRAF and MET. However, the potential relationships between ApoE and TSC2, ATRX, BRAF, and MET require further in—depth investigation.

The Apolipoprotein E knockout (ApoE^−/−^) mice model was employed to explore the role of ApoE in glioma. Specifically, we carried out in situ and subcutaneous tumour experiments in ApoE^−/−^ mice to monitor the tumour growth. The findings demonstrated that, in comparison to wild‐type (WT) mice, glioma growth was notably more rapid in ApoE knockout mice. This observation implies that the ApoE gene might exert an inhibitory effect on the regulating of glioma growth and dissemination. Researchers hypothesize that the absence of the ApoE gene could result in alterations in certain crucial signalling pathways or immune responses, consequently facilitating the accelerated growth of gliomas. Flow cytometry results indicated a decrease in CD8 T‐cell infiltration and a reduction in the secretion of granzyme and perforin in ApoE^−/−^ mice. Additionally, we examined myeloid cell markers and discovered an increase in myeloid‐derived suppressor cells (MDSC) in ApoE^−/−^ mice.

Through transcriptomic sequencing analysis of tumours in WT and ApoE^−/−^ mice, we observed that ApoE deficiency resulted in decreased expression levels of the immunologically relevant genes CCL24, CD74, and CX3CR1. The outcomes of KEGG and GO enrichment analyses disclosed that the absence of ApoE is intimately linked to the attenuation of immune‐related response pathways and immune molecule functions. Building on these findings, we postulate that the lack of ApoE might impair immune function, consequently diminishing the immunological activity of tumour‐infiltrating CD8 T cells.

Subsequently, we devised experiments wherein peritoneal macrophages were harvested from ApoE knockout mice and WT mice and co‐cultured with CD8 T cells. Our results indicated that the proportion of PD1‐positive and LAG3‐positive CD8 T cells, when stimulated by macrophages from ApoE knockout, was elevated. Concurrently, their capacity to secrete cytotoxic factors was diminished. These findings imply that macrophages lacking ApoE display a reduced ability to activate positive immune lymphocytes and an enhanced ability to activate negative immune lymphocytes.

We devised an experiment to extract peritoneal macrophages were isolated from ApoE‐knockout mice and wild‐type mice and then co‐cultured them with CD8 T cells. Our observations revealed that in the ApoE‐knockout group, there was an increase in the proportion of PD1‐positive and LAG3‐positive CD8 T cells, whereas the proportion of PERFORIN‐positive and GZMB‐positive CD8 T cells, which possess immune activity, decreased. These findings imply that ApoE‐deficient macrophages exhibit a decline in the activation of positive immune lymphocyte and an elevation in the activation of negative immune lymphocyte. Moreover, upon analysis using the online database Gliovis, we discovered a positive correlation between ApoE and PRF1, as well as between ApoE and GZMB, which further corroborates our hypothesis.

Our research emphasises the pivotal function of ApoE expression in suppressing glioma growth. This finding implies the possibility of devising new therapeutic strategies for glioma immunotherapy, especially in optimising adoptive cell therapy methods. By zeroing in on the ApoE pathway, we could potentially enhance the immune responses to glioma, presenting a fresh avenue for efficacious treatment modalities.

## Author Contributions

**Xiao‐fei Liu:** data curation (equal), methodology (equal), visualization (equal). **Yu‐jie Chang:** data curation (equal), visualization (equal). **Min Long:** formal analysis (equal), methodology (equal). **Kai‐qi Huang:** formal analysis (equal). **Bing Wang:** data curation (equal). **Duo Gong:** writing – original draft (lead). **Jun‐Li Luo:** writing – review and editing (equal). **Yong Feng:** project administration (lead), writing – review and editing (equal).

## Conflicts of Interest

The authors declare no conflicts of interest.

## Data Availability

Data sharing not applicable to this article as no datasets were generated or analysed during the current study.

## References

[1] L. M. Humphreys , P. Smith , Z. Chen , S. Fouad , and V. D'Angiolella , “The Role of E3 Ubiquitin Ligases in the Development and Progression of Glioblastoma,” Cell Death and Differentiation 28, no. 2 (2021): 522–537, 10.1038/s41418-020-00696-6. 33432111 PMC7862665

[2] K. Sadowski , A. Jażdżewska , J. Kozłowski , A. Zacny , T. Lorenc , and W. Olejarz , “Revolutionizing Glioblastoma Treatment: A Comprehensive Overview of Modern Therapeutic Approaches,” International Journal of Molecular Sciences 25, no. 11 (2024): 5774, 10.3390/ijms25115774. 38891962 PMC11172387

[3] Q. T. Ostrom , M. Price , C. Neff , et al., “CBTRUS Statistical Report: Primary Brain and Other Central Nervous System Tumors Diagnosed in the United States in 2016–2020,” Neuro‐Oncology 25 (2023): iv1–iv99, 10.1093/neuonc/noad149. 37793125 PMC10550277

[4] M. Price , C. Neff , N. Nagarajan , et al., “CBTRUS Statistical Report: American Brain Tumor Association & NCI Neuro‐Oncology Branch Adolescent and Young Adult Primary Brain and Other Central Nervous System Tumors Diagnosed in the United States in 2016–2020,” Neuro‐Oncology 26 (2024): iii1–iii53, 10.1093/neuonc/noae047. 38709657 PMC11073545

[5] J. E. Eckel‐Passow , D. H. Lachance , A. M. Molinaro , et al., “Glioma Groups Based on 1p/19q, IDH, and TERT Promoter Mutations in Tumors,” New England Journal of Medicine 372, no. 26 (2015): 2499–2508, 10.1056/NEJMoa1407279. 26061753 PMC4489704

[6] S. Rajendran , Y. Hu , A. Canella , et al., “Single‐Cell RNA Sequencing Reveals Immunosuppressive Myeloid Cell Diversity During Malignant Progression in a Murine Model of Glioma,” Cell Reports 42, no. 3 (2023): 112197, 10.1016/j.celrep.2023.112197. 36871221

[7] A. Vidyarthi , T. Agnihotri , N. Khan , et al., “Predominance of M2 Macrophages in Gliomas Leads to the Suppression of Local and Systemic Immunity,” Cancer Immunology, Immunotherapy: CII 68, no. 12 (2019): 1995–2004, 10.1007/s00262-019-02423-8. 31690954 PMC11028103

[8] J. Godlewski , H. B. Newton , E. A. Chiocca , and S. E. Lawler , “MicroRNAs and Glioblastoma; the Stem Cell Connection,” Cell Death and Differentiation 17, no. 2 (2010): 221–228, 10.1038/cdd.2009.71. 19521422

[9] S. Liu , W. Wang , S. Hu , et al., “Radiotherapy Remodels the Tumor Microenvironment for Enhancing Immunotherapeutic Sensitivity,” Cell Death & Disease 14, no. 10 (2023): 679, 10.1038/s41419-023-06211-2. 37833255 PMC10575861

[10] A. M. Sonabend , A. Gould , C. Amidei , et al., “Repeated Blood‐Brain Barrier Opening With an Implantable Ultrasound Device for Delivery of Albumin‐Bound Paclitaxel in Patients With Recurrent Glioblastoma: A Phase 1 Trial,” Lancet Oncology 24, no. 5 (2023): 509–522, 10.1016/s1470-2045(23)00112-2. 37142373 PMC10256454

[11] A. Goenka , D. Tiek , X. Song , T. Huang , B. Hu , and S. Y. Cheng , “The Many Facets of Therapy Resistance and Tumor Recurrence in Glioblastoma,” Cells 10, no. 3 (2021): 484, 10.3390/cells10030484. 33668200 PMC7995978

[12] C. M. Kloske , C. J. Barnum , A. F. Batista , et al., “APOE and Immunity: Research Highlights,” Alzheimer's & Dementia: The Journal of the Alzheimer's Association 19, no. 6 (2023): 2677–2696, 10.1002/alz.13020. PMC13158998 36975090

[13] N. Pencheva , H. Tran , C. Buss , et al., “Convergent Multi‐miRNA Targeting of ApoE Drives LRP1/LRP8‐Dependent Melanoma Metastasis and Angiogenesis,” Cell 151, no. 5 (2012): 1068–1082, 10.1016/j.cell.2012.10.028. 23142051 PMC3753115

[14] B. N. Ostendorf , J. Bilanovic , N. Adaku , et al., “Common Germline Variants of the Human APOE Gene Modulate Melanoma Progression and Survival,” Nature Medicine 26, no. 7 (2020): 1048–1053, 10.1038/s41591-020-0879-3. PMC8058866 32451497

[15] M. H. Sherman , “Lipid Carriers in Cancer: Context Matters,” Cancer Research 81, no. 16 (2021): 4186–4187, 10.1158/0008-5472.Can-21-1930. 34400469

[16] Q. T. Ostrom , M. Price , C. Neff , et al., “CBTRUS Statistical Report: Primary Brain and Other Central Nervous System Tumors Diagnosed in the United States in 2015–2019,” Neuro‐Oncology 24, no. Suppl 5 (2022): v1–v95, 10.1093/neuonc/noac202. 36196752 PMC9533228

[17] A. C. Tan , D. M. Ashley , G. Y. López , M. Malinzak , H. S. Friedman , and M. Khasraw , “Management of Glioblastoma: State of the Art and Future Directions,” CA: A Cancer Journal for Clinicians 70, no. 4 (2020): 299–312, 10.3322/caac.21613. 32478924

[18] J. Kim , Y. Zhu , S. Chen , et al., “Anti‐Glioma Effect of Ginseng‐Derived Exosomes‐Like Nanoparticles by Active Blood‐Brain‐Barrier Penetration and Tumor Microenvironment Modulation,” Journal of Nanobiotechnology 21, no. 1 (2023): 253, 10.1186/s12951-023-02006-x. 37542285 PMC10401762

[19] Q. Cai , X. Li , H. Xiong , et al., “Optical Blood‐Brain‐Tumor Barrier Modulation Expands Therapeutic Options for Glioblastoma Treatment,” Nature Communications 14, no. 1 (2023): 4934, 10.1038/s41467-023-40579-1. PMC10427669 37582846

[20] S. Xu , L. Tang , X. Li , F. Fan , and Z. Liu , “Immunotherapy for Glioma: Current Management and Future Application,” Cancer Letters 476 (2020): 1–12, 10.1016/j.canlet.2020.02.002. 32044356

[21] F. Yasinjan , Y. Xing , H. Geng , et al., “Immunotherapy: A Promising Approach for Glioma Treatment,” Frontiers in Immunology 14 (2023): 1255611, 10.3389/fimmu.2023.1255611. 37744349 PMC10512462

[22] I. A. Windham and S. Cohen , “The Cell Biology of APOE in the Brain,” Trends in Cell Biology 34, no. 4 (2024): 338–348, 10.1016/j.tcb.2023.09.004. 37805344 PMC10995109

[23] G. S. Getz and C. A. Reardon , “Apoprotein E and Reverse Cholesterol Transport,” International Journal of Molecular Sciences 19, no. 11 (2018): 3479, 10.3390/ijms19113479. 30404132 PMC6275009

[24] S. Yu , L. Qian , and J. Ma , “Comprehensive Analysis of the Expression and Prognosis for APOE in Malignancies: A Pan‐Cancer Analysis,” Oncology Research 30, no. 1 (2022): 13–22, 10.32604/or.2022.026141. 37304007 PMC10207989

[25] H. Liu , Y. Sun , Q. Zhang , et al., “Pro‐Inflammatory and Proliferative Microglia Drive Progression of Glioblastoma,” Cell Reports 36, no. 11 (2021): 109718, 10.1016/j.celrep.2021.109718. 34525361

[26] X. Lin , J. Zhang , R. H. Zhao , W. J. Zhang , J. F. Wu , and G. Xue , “APOE Is a Prognostic Biomarker and Correlates With Immune Infiltrates in Papillary Thyroid Carcinoma,” Journal of Cancer 13, no. 5 (2022): 1652–1663, 10.7150/jca.63545. 35371313 PMC8965124

[27] B. Y. Nan , G. F. Xiong , Z. R. Zhao , X. Gu , and X. S. Huang , “Comprehensive Identification of Potential Crucial Genes and miRNA‐mRNA Regulatory Networks in Papillary Thyroid Cancer,” BioMed Research International 2021 (2021): 6752141, 10.1155/2021.6752141. 33521130 PMC7817291

[28] R. Wienecke , A. Guha , J. C. Maize, Jr. , R. L. Heideman , D. C. JE , and D. H. Gutmann , “Reduced TSC2 RNA and Protein in Sporadic Astrocytomas and Ependymomas,” Annals of Neurology 42, no. 2 (1997): 230–235, 10.1002/ana.410420215. 9266734

[29] A. J. Becker , M. Löbach , H. Klein , et al., “Mutational Analysis of TSC1 and TSC2 Genes in Gangliogliomas,” Neuropathology and Applied Neurobiology 27, no. 2 (2001): 105–114, 10.1046/j.0305-1846.2001.00302.x. 11437991

[30] P. Y. Wen , A. Stein , M. van den Bent , et al., “Dabrafenib Plus Trametinib in Patients With BRAF(V600E)‐Mutant Low‐Grade and High‐Grade Glioma (ROAR): A Multicentre, Open‐Label, Single‐Arm, Phase 2, Basket Trial,” Lancet Oncology 23, no. 1 (2022): 53–64, 10.1016/s1470-2045(21)00578-7. 34838156

[31] F. Cheng and D. Guo , “MET in Glioma: Signaling Pathways and Targeted Therapies,” Journal of Experimental & Clinical Cancer Research 38, no. 1 (2019): 270, 10.1186/s13046-019-1269-x. 31221203 PMC6585013

